# Pairing tones with vagus nerve stimulation improves brain stem responses to speech in the valproic acid model of autism

**DOI:** 10.1152/jn.00325.2024

**Published:** 2024-09-25

**Authors:** Yuko Tamaoki, Varun Pasapula, Tanya T. Danaphongse, Alfonso R. Reyes, Collin R. Chandler, Michael S. Borland, Jonathan R. Riley, Alan M. Carroll, Crystal T. Engineer

**Affiliations:** ^1^Texas Biomedical Device Center, The University of Texas at Dallas, Richardson, Texas, United States; ^2^School of Behavioral and Brain Sciences, The University of Texas at Dallas, Richardson, Texas, United States

**Keywords:** autism spectrum disorder, inferior colliculus, plasticity, vagus nerve stimulation, valproic acid

## Abstract

Receptive language deficits and aberrant auditory processing are often observed in individuals with autism spectrum disorders (ASD). Symptoms associated with ASD are observed in rodents prenatally exposed to valproic acid (VPA), including deficits in speech sound discrimination ability. These perceptual difficulties are accompanied by changes in neural activity patterns. In both cortical and subcortical levels of the auditory pathway, VPA-exposed rats have impaired responses to speech sounds. Developing a method to improve these neural deficits throughout the auditory pathway is necessary. The purpose of this study was to investigate the ability of vagus nerve stimulation (VNS) paired with sounds to restore degraded inferior colliculus (IC) responses in VPA-exposed rats. VNS paired with the speech sound “dad” was presented to a group of VPA-exposed rats 300 times per day for 20 days. Another group of VPA-exposed rats were presented with VNS paired with multiple tone frequencies for 20 days. The IC responses were recorded from 19 saline-exposed control rats and 18 VPA-exposed with no VNS, 8 VNS-speech paired VPA-exposed, and 7 VNS-tone paired VPA-exposed female and male rats. Pairing VNS with tones increased the IC response strength to speech sounds by 44% compared to VPA-exposed rats alone. Contrarily, VNS-speech pairing significantly decreased the IC response to speech compared with VPA-exposed rats by 5%. The present research indicates that pairing VNS with tones improved sound processing in rats exposed to VPA and suggests that auditory processing can be improved through targeted plasticity.

**NEW & NOTEWORTHY** Pairing vagus nerve stimulation (VNS) with sounds has improved auditory processing in the auditory cortex of normal-hearing rats and autism models of rats. This study tests the ability of VNS-sound pairing to restore auditory processing in the inferior colliculus (IC) of valproic acid (VPA)-exposed rats. Pairing VNS with tones significantly reversed the degraded sound processing in the IC in VPA-exposed rats. The findings provide evidence that auditory processing in autism rat models can be improved through VNS.

## Graphical abstract

**Figure:**
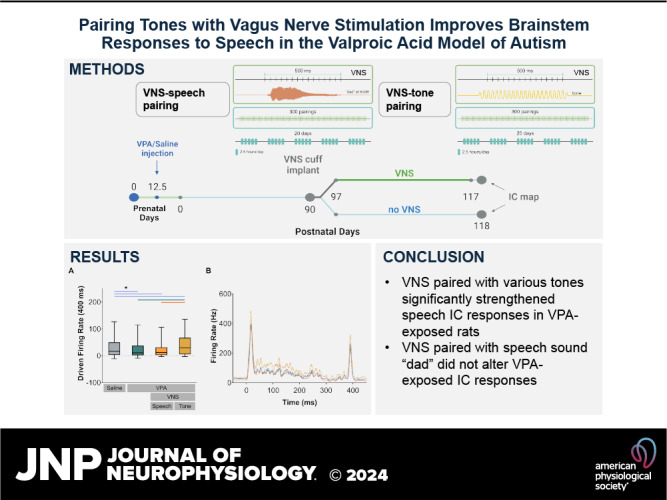

## INTRODUCTION

Autism spectrum disorder (ASD) is a neurodevelopmental disorder characterized by repetitive behaviors and impairments in social interactions and communication (1–4). Emerging evidence suggests that auditory processing deficits play a role in language and communication impairments (5–10).

Auditory evoked potential studies have revealed delayed and weakened responses to sounds in many children with ASD (7–13). Furthermore, ASD children with severe language impairments exhibit even greater delays in processing speech sounds (7–13). Given that auditory processing is often aberrant in children with autism, studies in rodent models of autism can provide further understanding of the underlying neural mechanisms that influence these degraded responses.

Valproic acid (VPA) is clinically used to treat epilepsy and bipolar disorder, and usage in pregnant women can increase the risk of autism in children when exposed in utero, with these children often presenting with impaired language development that is dose dependent (14–19). VPA-exposed rats exhibit ASD-related behavioral impairments, including social deficits, repetitive behaviors, and altered auditory behavioral performance (18, 20–25). For example, VPA-exposed rats performed significantly worse on auditory speech sound discrimination ability than saline-exposed control rats (21). Another study found that VPA-exposed rats were unable to discriminate different speeds of pulse trains (26). These auditory-related behavioral impairments in VPA-exposed rats are accompanied by abnormal neural auditory processing of sounds (26–32). In a previous study, VPA-exposed rats exhibited slower and weaker responses to speech sounds in the anterior auditory field (AAF) of the auditory cortex (27). Likewise, primary auditory cortex (A1) neurons in VPA-exposed rats were unable to process pulse trains at high repetition rates (26). VPA exposure effects are observed systemically throughout the auditory pathway. At the level of the brain stem, prenatal exposure to VPA alters neuronal morphology in areas such as the central nucleus of the inferior colliculus (CNIC) and ascending pathways to the medial geniculate nucleus (MGN) of the thalamus (30, 31, 33, 34). Because of these morphological changes, auditory processing in the inferior colliculus (IC) in these VPA-exposed rats was degraded (29, 32). Exposure to VPA significantly decreased the response strength to speech sounds in the inferior colliculus (32). Since VPA exposure impairs neural processing throughout the auditory pathway, the goal of the present study was to evaluate a potential therapy that can target specific plasticity to improve physiological responses to sounds in a rat ASD model.

Early intensive behavioral intervention (EIBI) is a behavioral therapy that is commonly used to improve behavioral challenges in individuals with ASD (12, 35–37). This behavioral intervention is started before the child reaches the age of 5 yr, and children attend these sessions for 20–40 h/wk for 1–2 yr (12, 35–37). Over the years, EIBI has provided an overall advancement in aiding children with autism (35, 36). However, individually, these children have variable responses to the therapy (33, 34). Despite the highly intensive treatment, ASD children with higher baseline cognitive function tend to benefit more than ASD children with lower baseline function (35, 36). Therefore, the need for adjunctive techniques that address individual differences and improve auditory processing more rapidly and to a greater extent in individuals with neurodevelopmental disorders is essential. One possibility is to manipulate the maladaptive physiological responses using vagus nerve stimulation (VNS).

Vagus nerve stimulation (VNS) releases plasticity-related neuromodulators, and when paired with sounds VNS-sound pairing can improve cortical responses to the paired sounds in typically hearing rats (38–42). Frequency-specific changes can be observed by pairing VNS with specific tones (38, 40). For example, when VNS was paired with a 9-kHz tone, the proportion of primary auditory cortex and the inferior colliculus that responds to the paired tone frequency is increased (38, 40). Furthermore, pairing tone trains with VNS can direct temporally specific plasticity in the auditory cortex (42). When VNS was paired with faster or slower tone trains, the auditory cortex response strength to rapid sounds increased or decreased (42). Additionally, VNS paired with speech sounds promoted spectrotemporally specific plasticity, increasing cortical responses to the paired speech sounds (39). Therefore, VNS-sound pairing can evoke plasticity that is specific to the paired sound and may benefit the degraded auditory processing observed in rat models of autism (38, 39). Previously, researchers tested VNS-sound pairing in a rat model of Rett syndrome and found that pairing VNS with tones reversed maladaptive plasticity in the auditory cortex (43). Based upon the success of these previous studies, the objective of the present study is to test the ability of VNS paired with sounds to enhance the degraded sound processing in the inferior colliculus of prenatally VPA-exposed rats.

## METHODS

A total of 33 VPA-exposed rats (17 females and 16 males) and 19 saline-exposed rats (9 females and 10 males) were used for this study. Of the 33 VPA-exposed rats, 8 VPA-exposed rats were randomly assigned to receive 20 days of VNS-speech pairing and 7 were randomly assigned to receive 20 days of VNS-tone pairing. The University of Texas at Dallas Institutional Animal Care and Use Committee (protocol no. 18-07) approved all surgical and recording procedures.

### Animal Breeding

Pregnant Sprague Dawley rats were administered intraperitoneal injections of either 600 mg/kg of valproic acid (Sigma-Aldrich) dissolved in saline or 0.9% saline alone on embryonic day 12.5 (23, 24, 44, 45). The male and female Sprague Dawley rats used for breeding were obtained from Charles River Laboratory (Wilmington, MA). The 33 VPA-exposed rats came from nine different litters, and the 19 saline-exposed rats came from eight different litters.

### Vagus Nerve Stimulation Cuff Implantation

Once 90 days of age, 15 adult VPA-exposed rats were implanted with a bipolar cuff electrode around the left vagus nerve with a head cap that facilitated the connection to the stimulator. All implanted components for VNS were manufactured identically to the published protocol (46). VNS cuff implantation was performed under 3% isoflurane in oxygen as an inhalation anesthetic. A sagittal incision was made along the midline of the head, and the connective tissues between the left eye and ear were dissected to allow cuff leads to run subcutaneously. To implant the VNS cuff, the left vagus nerve was revealed with an incision above the left clavicle and by blunt dissection of the sternocleidomastoid and omohyoid muscles. The cuff was then enveloped and affixed around the nerve. Unilateral stimulation of the vagus nerve activates the central nervous system structures bilaterally (47, 48). Implantation of the head cap was done by placing the animal on a stereotaxic apparatus. After the connective tissues on top of the skull were removed, the head cap was secured along the anterior-posterior axis of the skull with a coating of acrylic. Incision sites were sutured, and Neosporin was applied topically immediately after the operation. Baytril tablets (2 mg/tablet) were given daily for 3 days postoperation (49, 50). All surgical procedures were identical to previous studies (38, 39, 43, 51).

### VNS-Speech Pairing

After a week of recovery, eight VPA-exposed rats received 300 bursts of vagus nerve stimulation per day temporally paired with the presentation of the speech word “dad” for a total of 20 days. The intensity of the speech sound was calibrated such that the loudest 100 ms was 60 dB SPL, and the speech sounds were shifted up one octave to better align with the rat’s audiogram. The stimulation and the sound were 500 ms long; both were presented simultaneously. The stimulation parameters consisted of 16 pulses with 100-µs biphasic pulse width at an intensity of 0.8 mA and a frequency of 30 Hz, as used in previous studies (38–40, 43, 52–54). Twenty-four hours after the 20th day of VNS-sound pairing, the rats underwent inferior colliculus recording.

### VNS-Tone Pairing

Similar to VNS-speech paired VPA-exposed rats, seven of the VPA-exposed rats received 20 days of vagus nerve stimulation paired with presentation of multiple tones. The stimulation parameters were identical to the VNS-speech paradigm. The stimuli consisted of tones with frequencies spanning the rat hearing range (1.3, 2.2, 3.7, 6.3, 10.6, 17.8, and 29.9 kHz), with the intensity for each tone frequency selected to match the contour of the rat audiogram (55, 50, 40, 35, 35, 45, and 55 dB, respectively). These tones were presented so the tone frequency was randomly interleaved between trials, and the tone was simultaneously presented with the stimulation, identical to a previous study (43). After the last session of VNS-tone pairing, the inferior colliculus responses of these rats were recorded.

### Physiological Recordings

In vivo extracellular multiunit recordings were collected from the right central nucleus of the inferior colliculus. A total of 453 sites from saline-exposed rats (*n* = 19), 374 sites from VPA-exposed rats without VNS therapy (*n* = 18), 183 sites from VNS-speech paired VPA-exposed rats (*n* = 8), and 146 sites from VNS-tone paired VPA-exposed rats (*n* = 7) were obtained. The IC responses from saline-exposed rats and VPA-exposed rats without VNS therapy have been previously published (32). The IC recordings of the rats from all four groups of animals were interleaved in time. All recordings were performed with the experimenters blind to the experimental group. Rats were administered an intraperitoneal injection of 50 mg/kg of sodium pentobarbital for anesthesia. Supplemental doses of pentobarbital (8 mg/mL) were given as needed. A tracheotomy was performed to maintain breathing. Consistent with previous studies, responses from the central nucleus of the inferior colliculus (CNIC) were recorded with two parylene-coated tungsten microelectrodes (1–2 MΩ, 250 µm apart; FHC) that were lowered through a hole drilled in the skull 9 mm posterior from the bregma and 1.5 mm lateral from the midline suture above the right inferior colliculus at 200-µm intervals ventrally from a depth of 1,000 µm below the surface of the brain to 5,000 µm (32, 38, 55, 56). All recordings were completed in a soundproofed chamber with a Tucker Davis Technologies (TDT) MF-1 speaker placed 10 cm from the animal’s left ear, and recordings were obtained from the contralateral side. At each recording site, 1,296 randomly interleaved tones were each 25 ms long with a 5-ms cosine ramp on tone onset and offset. The band pass ranged from 300 to 3,000 Hz, and the sampling rate was set to 25 kHz. Tones consisting of 81 frequencies distributed logarithmically from 1 to 32 kHz, with increments of 0.0625 octaves, across 16 intensities ranging from 0 to 75 dB SPL, in 5-dB increments, were presented. Furthermore, speech sounds (“chad,” “dad,” “deed,” “dood,” “fad,” “gad,” “had,” “jad,” “sad,” “shad,” “tad”) were randomly interleaved 20 times each (Supplemental Fig. S1). All speech sounds were calibrated to be 60 dB at the loudest 100 ms of the vowel portion of the sound. Six 25-ms-long white noise burst trains at 10 Hz were also randomly interleaved 20 times. Sensory electrophysiology hardware (RZ5) and software (BrainWare; TDT) were used to acquire multiunit neural activity.

To confirm that the recordings were taken from the central nucleus of the IC, a number of factors were taken into account. By progressing the microelectrodes dorsal-ventrally, we were able to obtain a clear tonotopic organization that distinguishes the central nucleus of the IC from other areas outside the region of interest, as supported by previous studies (38, 55–59). Comparing the percentage of inferior colliculus sites responding to each tone frequency, VNS-sound pairing did not alter the tonotopic organization among experimental groups and no differences were found in the percentage of sites responding to tones in each of five 1-octave frequency bins spanning from 1 to 32 kHz (Kruskal–Wallis, *P* = 0.95; Supplemental Fig. S2). Continuous correlation between the characteristic frequency and recording depth was used to confirm that the analysis consisted of recordings from the central nucleus of the IC (Supplemental Fig. S3).

### Data Analysis

All data analyses were conducted with custom MATLAB software and GraphPad Prism 10.2.2. Driven firing rate evoked from 400-ms, 40-ms, and 300-ms durations of the sound were used to determine the response strength of the whole speech and consonant portion and vowel portion of the speech sound, respectively.

To determine the neural discrimination accuracy between sound pairs, a nearest-neighbor classifier was used (55, 60, 61). For each sound pair (example: “dad” vs. “bad”), peristimulus time histogram (PSTH) templates of the IC response were constructed from 19 of the 20 repeats recorded for each sound. The remaining single-trial repeat for each sound was then compared to each template and quantified as the Euclidean distance between each single-trial response to each of the templates. That single-trial response was assigned to the sound template that evoked the minimum Euclidian distance (most similar response). The consonant pairs were selected to include speech sounds differing solely in the initial consonant portion of the sound (forty 1-ms bins of the first 40 ms of IC response) and the vowel pairs compared the speech sounds differing in the vowel portion of the sound (“dad” vs. “deed” vs. “dood”; single 300-ms bin beginning at initial consonant offset).

Total extracellular spikes within the first 32 ms following tone onset was calculated to determine tone response strength. Additionally, receptive field properties were computed for the tone responses, including response threshold, bandwidth 10 dB and 40 dB above the threshold, onset latency, peak latency, end of peak latency, and spontaneous firing rate, as in previous studies (27, 38, 43, 53, 54, 62). For noise burst analysis, the average number of driven spikes from all six bursts was calculated to determine the firing rate. Onset latency was determined as the latency of the first spike onset after sound presentation. Peak latency was quantified as the timing at the maximum firing rate from the first peak. The synchronicity of noise bursts was also determined using vector strength and Rayleigh statistics. All noise burst analyses were identical to previous studies (32, 55, 61, 63, 64).

The Kolmogorov–Smirnov test was used to test normality. Since the population for each group was not normally distributed for all analyses, a nonparametric Mann–Whitney *U* test was used to compare responses between groups. To compare differences among all groups, a Kruskal–Wallis test was implemented.

## RESULTS

### Selective Improvements to Speech Sound Processing after VNS-Sound Pairing

VPA-exposed rats exhibit significantly weaker responses to speech sounds in the anterior auditory field and the inferior colliculus compared to saline-exposed rats, and restoring this response could improve auditory processing (27, 32). In this study, we tested whether VNS-sound pairing would improve the degraded inferior colliculus responses and whether pairing tones or speech sounds would promote greater plasticity. After 20 days of VNS-speech pairing or VNS-tone pairing, multiunit IC responses were recorded. Responses to speech sounds, tones, and noise burst trains were compared between experimental groups.

**Figure 1.**
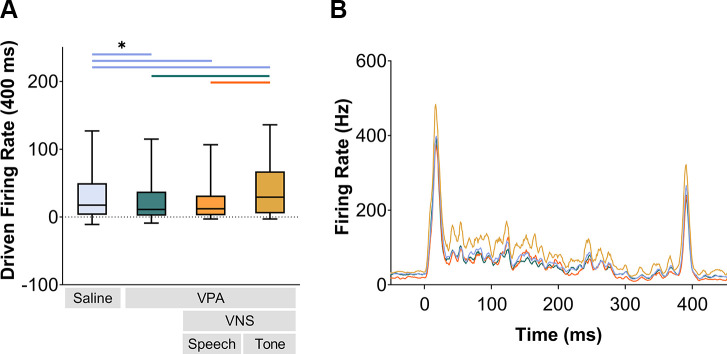
Vagus nerve stimulation (VNS)-tone pairing increased the inferior colliculus (IC) response strength to speech sounds. *A*: the driven firing rate to the 400-ms duration of the speech sounds was strongest in VNS-tone paired valproic acid (VPA)-exposed rats. The error bars indicate 95% confidence interval, and the bar in the box represents the median. Significance was determined through group comparisons using Mann–Whitney *U* tests and is represented by colored lines and an asterisk (*). The line colors denote the groups being compared. *B*: peristimulus time histogram (PSTH) evoked in response to speech sound “dad.” (Saline *n* = 453 sites; VPA *n* = 374 sites; VNS-speech *n* = 183 sites; VNS-tone *n* = 146 sites.)

**Figure 2.**
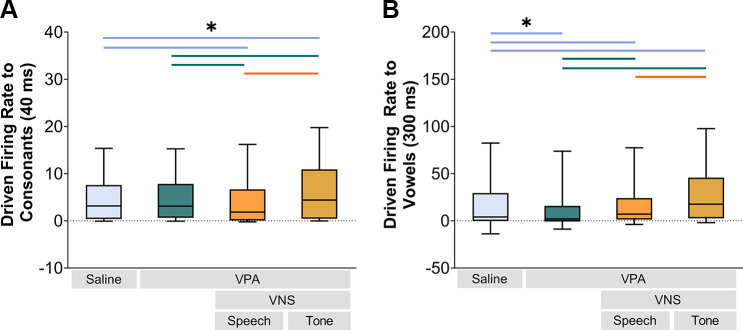
Vagus nerve stimulation (VNS)-sound pairing altered inferior colliculus (IC) responses to the consonant and vowel portions of the speech sounds. *A*: VNS-tone pairing in valproic acid (VPA)-exposed rats evoked the strongest driven response to the initial consonant portion of the sound (1st 40 ms). *B*: both of the VNS-sound pairing groups increased the driven firing rate responses to the vowel portion of the sounds. The line in the box and the error bars represent median and 95% confidence interval, respectively. The colored lines and the asterisk (*) indicate significant differences between groups which were calculated with Mann–Whitney *U* test. The line colors denote the groups being compared. (Saline *n* = 453 sites; VPA *n* = 374 sites; VNS-speech *n* = 183 sites; VNS-tone *n* = 146 sites.)

Comparing responses to the whole 400-ms duration of the speech sounds, a significant difference in response strength was observed across experimental groups (Kruskal–Wallis, *P* < 0.001). VPA exposure significantly reduced the response strength to speech sounds in the inferior colliculus compared to saline-exposed control rats (Mann–Whitney *U*, *P* < 0.0001). VNS-tone pairing significantly strengthened the response strength to speech sounds in the inferior colliculus in VPA-exposed rats (Mann–Whitney *U*, *P* < 0.0001), surpassing the driven firing rate of the saline-exposed rats (Mann–Whitney *U*, *P* < 0.0001; Fig. 1, *A* and *B*). Interestingly, the VPA-exposed rats that received 20 days of VNS-speech pairing did not exhibit a strengthened IC response to speech sounds compared to VPA-exposed rats alone (Mann–Whitney *U* test, *P* = 0.35; Fig. 1*A*). Since the speech sounds contain both consonants and vowels, we evaluated whether the response strength difference was confined to either the onset (consonant) or the sustained (vowel) portion of the sound. Both components of the response differed across groups (Kruskal–Wallis, Consonant: *P* < 0.001; Vowel: *P* < 0.001; Fig. 2, *A* and *B*). VNS-tone pairing significantly increased the IC driven firing rate to both the consonant and vowel portions of the sound compared to VPA-exposed rats alone (Mann–Whitney *U*, *P* < 0.001; Fig. 2, *A* and *B*). VNS-speech pairing decreased the IC response strength to the consonant portion of the sound (Mann–Whitney *U*, *P* < 0.001; Fig. 2*A*) but increased the IC response strength to the vowel portion of the sound (Mann–Whitney *U*, *P* < 0.001; Fig. 2*B*). These results suggest that the specific sounds used in conjunction with VNS can modulate the degraded responses in VPA-exposed rats, which is consistent with earlier reports that pairing different sounds with VNS produces distinct forms of plasticity (38–42).

**Figure 3.**
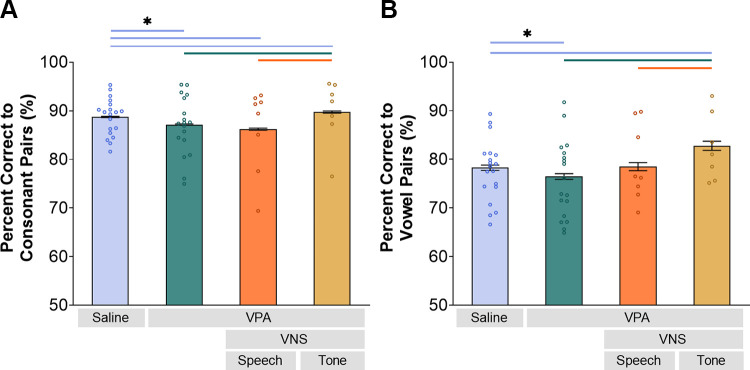
Vagus nerve stimulation (VNS)-tone pairing enhanced neural discrimination ability in valproic acid (VPA)-exposed rats. *A*: the neural classifier accuracy to pairs of sounds differing in initial consonant (D-, G-, T-, Ch-, J-, S-, Sh-, F-, H-) was compared between groups. VNS-tone pairing in VPA-exposed rats significantly increased the neural discrimination accuracy (Mann-Whitney *U*, *P* < 0.0001). *B*: VNS-tone pairing also significantly increased neural classifier accuracy to pairs of sounds differing in vowel (“Dad,” “Deed,” “Dood”) in VPA-exposed rats (Mann–Whitney *U*, *P* < 0.0001). The bars represent the mean neural discrimination accuracy. The error bars are SE across recording sites. The colored lines and the asterisk (*) indicate significant differences between groups. The circles represent each individual animal’s average neural classifier accuracy. (Saline *n* = 453 sites; VPA *n* = 374 sites; VNS-speech *n* = 183 sites; VNS-tone *n* = 146 sites.)

To evaluate whether these changes improved the ability of neurons to discriminate between similar speech sounds, we compared performance of a neural classifier provided with activity from each of the experimental groups. There was a significant difference in neural classifier performance across experimental groups (Kruskal–Wallis, Consonant: *P* < 0.001; Vowel: *P* < 0.001). Compared to saline-exposed rats, VPA-exposed rats had significantly less accurate performance in discriminating sound pairs differing in both the initial consonant and the vowel (Consonant: Mann–Whitney *U*, *P* < 0.0001; Vowel: Mann–Whitney *U*, *P* < 0.0001; Fig. 3, *A* and *B*). In comparison to VPA-exposed rats without VNS, VNS-tone paired VPA-exposed rats had significantly more accurate performance in discriminating sound pairs differing in both the initial consonant and the vowel (Consonant: Mann–Whitney *U*, *P* < 0.0001; Vowel: Mann–Whitney *U*, *P* < 0.0001; Fig. 3, *A* and *B*). VNS-speech pairing did not alter the neural classifier performance compared to VPA-exposed rats without VNS for consonant pairs (Mann–Whitney *U*, *P* = 0.12; Fig. 3*A*) or vowel pairs (Mann–Whitney *U*, *P* = 0.085; Fig. 3*B*). These results suggest that VNS-tone pairing was able to enhance degraded IC speech processing in VPA-exposed rats.

### Tone Responses Remain Unaltered after VNS-Sound Pairing

**Figure 4.**
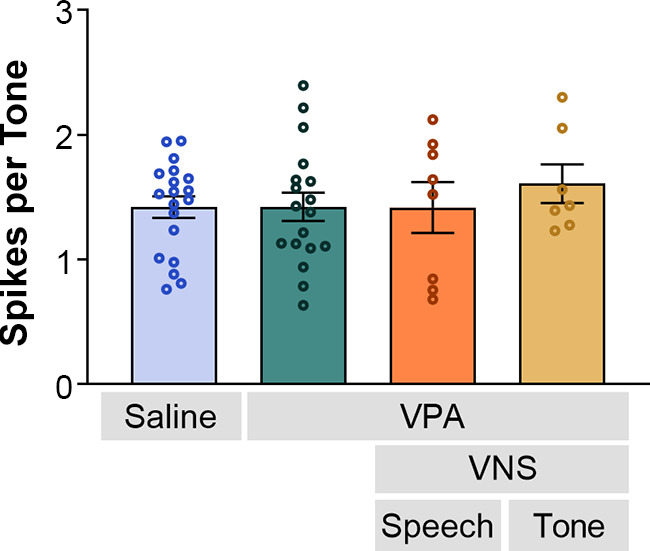
Vagus nerve stimulation (VNS)-sound pairing did not significantly alter the inferior colliculus (IC) response strength to tones (Kruskal–Wallis, *P* = 0.90). Mean spikes per tone and SE were calculated across recording sites and are indicated as bars and error bars, respectively (saline *n* = 453 sites; VPA *n* = 374 sites; VNS-speech *n* = 183 sites; VNS-tone *n* = 146 sites). Each animal’s average spikes per tone are shown as circles (saline *n* = 19; VPA *n* = 18; VNS-speech *n* = 8, VNS-tone *n* = 7).

Compared to saline-exposed rats, VPA-exposed rats do not exhibit response differences to tones in the inferior colliculus (32). However, we hypothesized that VNS-tone paired animals would exhibit increased IC responses to tones, as observed in previous studies in typically hearing rats (38). In this study, there were no significant differences in spikes evoked by tones among groups (Kruskal–Wallis, *P* = 0.90). VNS-speech pairing and VNS-tone pairing did not change the VPA-exposed rats’ IC responses to tones (Mann–Whitney *U*, VNS-speech: *P* = 0.89; VNS-tone: *P* = 0.39; Fig. 4). Additionally, no significant differences were observed in response threshold, bandwidth, onset and peak latency, end of peak latency, and spontaneous firing rates among the groups [Brown–Forsythe ANOVA; threshold: *F*(3,35.80) = 1.05, *P* = 0.38; bandwidth at 10 dB: *F*(3,39.01) = 0.60, *P* = 0.62; bandwidth at 40 dB: *F*(3,31.52) = 0.22, *P* = 0.88; onset latency: *F*(3,21.76) = 0.62, *P* = 0.61; peak latency: *F*(3,27.58) = 0.61, *P* = 0.61; end of peak latency: *F*(3,32.42) = 0.11, *P* = 0.95; and spontaneous firing rate: *F*(3,21.46) = 0.40, *P* = 0.75; Table 1]. Overall, VPA alters the neural plasticity produced by VNS pairing.

**Table 1.** Threshold, bandwidth 10 and 40 dB above threshold, onset and peak latency, end of peak latency, and spontaneous firing rates were not altered after VNS-sound pairing

	Saline	VPA	VPA + VNS Speech	VPA + VNS Tone
Threshold, dB	17.35 ± 1.61	17.35 ± 2.29	17.78 ± 2.95	12.35 ± 2.19
Bandwidth 10 dB, octaves	1.33 ± 0.13	1.54 ± 0.18	1.28 ± 0.20	1.32 ± 0.19
Bandwidth 40 dB, octaves	1.73 ± 0.19	1.90 ± 0.19	1.85 ± 0.31	1.97 ± 0.32
Latency, ms	8.21 ± 0.19	8.47 ± 0.31	8.29 ± 0.36	7.71 ± 0.60
Peak latency, ms	13.38 ± 0.21	13.20 ± 0.49	13.01 ± 0.62	12.37 ± 0.69
End of peak, ms	27.11 ± 0.30	26.93 ± 0.81	27.32 ± 0.77	26.66 ± 0.87
Spontaneous firing rate, Hz	30.68 ± 6.05	30.82 ± 6.48	21.96 ± 3.74	36.31 ± 12.79

Values represent the mean taken across all recording sites ± SE. VNS, vagus nerve stimulation; VPA, valproic acid.

### VNS Pairing Alters Noise Burst Responses

Difficulties in speech perception that are often observed in individuals with ASD have been associated with deficits in processing rapid temporal sound changes (9, 10, 28, 65). This observation has also been observed in VPA-exposed rats, in which weaker and asynchronous responses to noise bursts were observed in the anterior auditory field (AAF) (27). However, in the inferior colliculus, VPA-exposed rats do not have impaired responses to a slow 10-Hz train of noise bursts (32).

**Figure 5.**
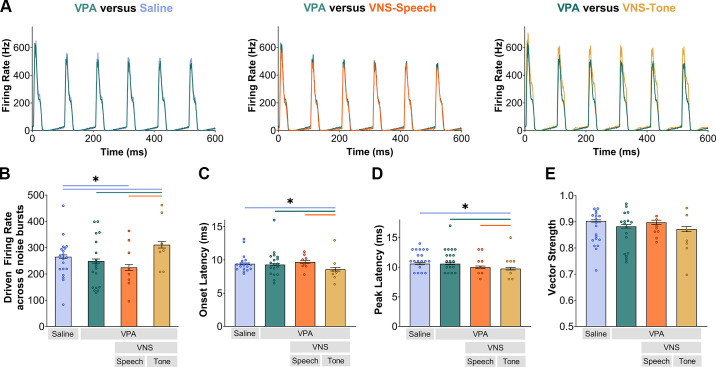
Vagus nerve stimulation (VNS)-tone pairing significantly altered response strength and timing to noise bursts. *A*: peristimulus time histograms (PSTH) of a train of 6 noise bursts presented at 10 Hz comparing valproic acid (VPA) to other experimental groups. *Left*: PSTH comparing VPA- to saline-exposed rats. *Center*: PSTH of VPA in comparison to VNS-speech paired VPA-exposed rats. *Right*: PSTH represents VPA-exposed rats compared to VNS-tone paired group. *B*: VNS-tone paired VPA-exposed rats had increased driven firing rate across all 6 noise bursts compared to VPA-exposed rats, saline-exposed rats, and VNS-speech paired VPA-exposed rats. VNS-speech pairing, on the other hand, significantly reduced the response strength. *C*: the timing from the onset of the noise burst train was significantly reduced after 20 days of VNS-tone pairing. *D*: VNS-tone pairing also decreased the peak latency from the first peak. *E*: all experimental groups had synchronous responses to all 6 noise bursts. For all panels, the bars and error bars are mean and SE, respectively, across recording sites (saline *n* = 453 sites; VPA *n* = 374 sites; VNS-speech *n* = 183 sites; VNS-tone *n* = 146 sites). Each circle represents average individual animal data (saline *n* = 19; VPA *n* = 18; VNS-speech *n* = 8; VNS-tone *n* = 7). Significance between groups is indicated with colored lines and an asterisk (*) and was determined with Mann–Whitney *U* tests.

Since VNS-sound pairing can alter spectral and temporal aspects of sound processing, the driven firing rate, onset and peak latency, and vector strength to a train of six noise bursts presented at a rate of 10 Hz were compared between experimental groups. Comparing all groups, the peak firing rate across all the noise bursts was significantly different (Kruskal–Wallis, *P* < 0.001; Fig. 5, *A* and *B*). VNS-tone paired rats responded significantly stronger to all six noise bursts compared to saline-exposed rats (Mann–Whitney *U*, *P* < 0.001; Fig. 5*B*), VPA-exposed rats without VNS (Mann–Whitney *U*, *P* < 0.001; Fig. 5, *A* and *B*), and VNS-speech paired VPA-exposed rats (Mann–Whitney *U*, *P* < 0.001; Fig. 5*B*). Interestingly, VNS-speech pairing significantly decreased the driven firing rate compared to saline-exposed rats (Mann–Whitney *U*, *P* < 0.001; Fig. 5*B*). Onset and peak latency were also significantly different among the experimental groups (Kruskal–Wallis, Onset: *P* = 0.005; Peak: *P* < 0.001; Fig. 5, *C* and *D*). For the onset timing of the noise bursts, VNS-tone paired VPA-exposed rats responded significantly faster than saline-exposed (Mann–Whitney *U*, *P* = 0.003), VPA-exposed (Mann–Whitney *U*, *P* = 0.016), and VNS-speech paired VPA-exposed (Mann–Whitney *U*, *P* < 0.001) rats. VNS-tone pairing also significantly decreased the peak response latency to the first noise burst in comparison to both saline-exposed rats (Mann–Whitney *U*, *P* < 0.001) and VPA-exposed (Mann–Whitney *U*, *P* < 0.001, Fig. 5*D*) rats. Meanwhile, VNS-speech pairing did not alter the latency responses to noise bursts compared to VPA-exposed rats (Mann–Whitney *U*, Onset: *P* = 0.14, Peak: *P* = 0.06; Fig. 5, *C* and *D*). VNS-sound pairing did not alter vector strength compared to VPA-exposed rats alone (Mann–Whitney *U*, VNS-speech: *P* = 0.37; VNS-tone: *P* = 0.06; Fig. 5*E*). Noise burst responses were significantly phase locked in all experimental groups (all Rayleigh statistics > 13.8). Overall, VNS-tone pairing in VPA-exposed rats promoted stronger and faster responses to sounds with rapid temporal changes.

## DISCUSSION

Receptive language deficits observed in individuals with autism spectrum disorder are associated with abnormal auditory processing (7–11, 13, 17, 28, 66). Auditory evoked potentials reveal delayed and weakened responses from children with ASD and rat models (7–11, 18, 22–25, 32, 67). Previous studies have documented that these VPA-exposed rats have altered physiological responses in several auditory processing areas (26, 27, 32). Therefore, the focus of this study was to test the ability of VNS paired with sounds to improve the impaired inferior colliculus responses to sounds that were observed in VPA-exposed rats.

### Synaptic Plasticity in ASD

The abnormal behaviors observed in ASD can be rooted in influences from multiple factors. Studies have speculated that these phenotypical characteristics of ASD are driven by physiological dysfunction at the level of neural circuits (68–71). For example, excess excitatory circuits are postulated to be the driving factor behind hyperexcitability observed in ASD (68, 70). In VPA-exposed rats, previous studies have documented reduced numbers of parvalbumin-positive cells (PV-cells), which decreases inhibitory circuitry and increases excitation, creating an imbalanced excitation-inhibition ratio (23, 72, 73). Additionally, prenatal exposure to VPA alters the expression of proteins involved in central nervous system (CNS) development (74, 75). Prenatally exposed VPA rats had less mRNA expression of brain-derived neurotrophic factor (BDNF) and increased NMDA receptors in several regions of the brain (73, 74, 76). Since VPA exposure alters synaptic plasticity at the cellular and molecular levels, restoring this may provide better outcomes cortically and behaviorally. Previous studies have found that VNS paired with activities, such as tactile rehabilitation, alters the level of expression of Activity-regulated cytoskeleton-associated protein (Arc), a protein that plays a role in synaptic plasticity, in the cortex (77–79). The effects seen in this study, in which VNS enhanced synaptic plasticity in VPA-exposed rats, may have resulted from VNS promoting molecular signaling changes (41, 80, 81).

### VNS-Sound Pairing in VPA-Exposed Rats

Pairing vagus nerve stimulation with sound results in enhancement of cortical responses in multiple domains (38–41, 61, 82). A previous study found that pairing VNS with a 9-kHz tone significantly strengthened the neural response to tones in the A1 as well as the IC, suggesting that VNS-tone pairing can effectively enhance auditory processing spectrally (38). VNS-tone pairing is effective at reversing the maladaptive plasticity in a noise-induced rat model of tinnitus (40). After pairing VNS with multiple tones that spanned the rats’ hearing range, the degraded physiological responses observed in the tinnitus model were restored to preinjury levels (40). Furthermore, VNS-tone pairing improved the auditory cortex responses in a rat model of Rett syndrome (43). These VNS treatment effects are expected to be long-lasting based on previous preclinical (3–7 wk after VNS) and clinical (3 mo to 3 yr) studies (40, 83–85). Therefore, the present study examined whether VNS paired with tones that spanned the hearing range of a rat can reverse the maladaptive plasticity in rats prenatally exposed to VPA. Parallel to the previous observations, VNS-tone pairing was able to restore the degraded IC responses to speech sounds in VPA-exposed rats.

In addition to tone pairing, VNS has also amplified cortical responses when paired with speech sounds in typically hearing rats (39, 82). By pairing VNS with speech words “rad” and “lad,” normal-hearing rats had increased A1 response strength and neural discrimination accuracy to the paired sounds (39, 82). Although VNS-speech pairing strengthened neural responses in the auditory cortex, whether this was observed in the inferior colliculus and if VNS-speech pairing can reverse pathological plasticity was still unknown. Therefore, the present study sought to investigate this by pairing VNS with the speech sound “dad” for 20 days in another group of VPA-exposed rats. Contrary to previous findings, VNS-speech pairing was not able to improve speech responses in the IC in VPA-exposed rats. One possibility for this finding could lie in the time course of plasticity. The time course of plasticity in cortical versus subcortical auditory structures is known to be different in typically hearing animals, where A1 plasticity is typically larger and longer-lasting than IC plasticity (86). Interestingly, we have previously documented that 20 days of VNS-tone pairing evokes comparable response changes in both A1 and IC in typically hearing rats (38). In the present study, we found that VNS-tone pairing, but not VNS-speech pairing, enhanced IC responses to sounds in VPA-exposed rats. One possibility is that VNS-speech pairing could have a smaller or shorter-lasting plasticity effect than VNS-tone pairing, in which case A1 responses might be different from IC responses following VNS pairing in VPA-exposed rats. Future studies are needed to explore the time course of changes across the auditory pathway following VNS-sound pairing in VPA-exposed rats.

The findings from the present study were able to identify that VNS-tone pairing improved the inferior colliculus response to sounds and lay the groundwork for future studies that can explore the ability of VNS-sound pairing therapy for clinical populations. First, the implications of pairing VNS with tones for perceptual ability should be evaluated. Additionally, future studies should optimize the VNS therapy that can maximize beneficial outcomes.

### Narrowband versus Broadband Sound Processing in IC

The inferior colliculus plays a crucial role in processing auditory information. It integrates both spectral and temporal characteristics of sound, allowing for a comprehensive interpretation of pitch discrimination (55, 58, 87, 88). This duality is important for processing broadband stimuli like speech, where the frequency and timing of the sounds help distinguish the different components of a speech sound (55, 88). Alternatively, processing narrowband stimuli like tone bursts with no changes in pitch does not require temporal information to process the sound (88). Through studies with VPA-exposed rats, broadband processing has been shown to be impaired (29, 32). Malhotra and Kulesza (29) found that VPA-exposed rats had degraded auditory brain stem responses (ABRs) to broadband click sounds. In addition to degraded ABRs, the inferior colliculus of VPA-exposed rats also showed degraded speech responses but intact tone and noise burst responses (32). Since the deficits were specific to complex speech sound processing, the findings from these studies suggest that the brain stem, specifically the inferior colliculus, in these VPA-exposed rats has difficulty processing temporal cues that exist in complex sounds like speech. Additionally, previous studies have found that VPA exposure decreases connections from the cochlear nucleus to IC, which may affect frequency coding or other aspects of auditory processing (89). The present study highlighted the discrepancy between inferior colliculus responses after VNS-tone pairing and VNS-speech pairing. A possible reasoning behind the differences may be the VPA exposure affecting the inferior colliculus by impairing temporal cue processing necessary for sounds with rapid spectrotemporal changes. Another mechanism behind these differences in responses may also be that VPA exposure reduces habituation, another common observation in ASD. With the inability to habituate to sounds, children with ASD respond with difficulty to complex sounds made up of multiple acoustic components while responding typically to more simple sounds (11). Similarly, in these VPA-exposed rats, responses to speech sounds were degraded while responses to tones remained unimpaired in the IC.

### Clinical Issues in the IC

In auditory brain stem response studies, children with ASD often exhibited atypical responses to sounds containing pitch variations (9–11, 13, 66, 90, 91). For example, when comparing brain stem responses from click stimuli and speech sounds, children with ASD had normal click-evoked responses, but an aberrant speech-evoked response was observed (9, 10). Overall, in both rodent models and humans with autism, the brain stem responses show greater deficits toward broadband stimuli like speech sounds. Based on the present findings, VNS-tone pairing was able to improve the degraded speech processing in VPA-exposed rats. We envision that it would be straightforward to integrate VNS-sound pairing into the current behavioral intervention already being used for early-age children with autism, EIBI, to improve auditory perception in children with ASD. During therapy, the therapist can push a button to stimulate the vagus nerve every time the child hears a sound, similar to the VNS-movement pairing therapy that is currently used to improve upper limb function in individuals after a stroke (83, 92, 93). As a next step, optimizing the VNS-sound pairing paradigm to improve the auditory processing in the brain stem could potentially lead to significant improvement in language and communication abilities for those with ASD (9, 10).

## DATA AVAILABILITY

Data will be made available upon reasonable request.

## SUPPLEMENTAL MATERIAL

10.6084/m9.figshare.27018310 Supplemental Fig. S1: https://doi.org/10.6084/m9.figshare.27018310.

10.6084/m9.figshare.27018313 Supplemental Fig. S2: https://doi.org/10.6084/m9.figshare.27018313.

10.6084/m9.figshare.27018316 Supplemental Fig. S3: https://doi.org/10.6084/m9.figshare.27018316.

## GRANTS

This work was supported by a grant from the National Institutes of Health to C.T.E. (Grant No. R01DC017480).

## DISCLOSURES

C. T. Engineer is married to an employee of MicroTransponder Inc. None of the other authors has any conflicts of interest, financial or otherwise, to disclose.

## AUTHOR CONTRIBUTIONS

Y.T. and C.T.E. conceived and designed research; Y.T., V.P., T.T.D., A.R.R., C.R.C., and M.S.B. performed experiments; Y.T., M.S.B., J.R.R., and A.C. analyzed data; Y.T., A.M.C., and C.T.E. interpreted results of experiments; Y.T. prepared figures; Y.T. drafted manuscript; Y.T., T.T.D., M.S.B., J.R.R., A.M.C., and C.T.E. edited and revised manuscript; Y.T., V.P., T.T.D., A.R.R., C.R.C., M.S.B., J.R.R., A.M.C., and C.T.E. approved final version of manuscript.
